# Effects of Exercise Interventions on Immune Function in Children and Adolescents With Cancer and HSCT Recipients - A Systematic Review

**DOI:** 10.3389/fimmu.2021.746171

**Published:** 2021-09-27

**Authors:** Ronja Beller, Sabrina Bianca Bennstein, Miriam Götte

**Affiliations:** ^1^ Department of Pediatric Hematology/Oncology, Center for Child and Adolescent Medicine, Clinic for Pediatrics III, West German Cancer Centre, University Hospital Essen, Essen, Germany; ^2^ Institute for Transplantation Diagnostics and Cell Therapeutics, Medical Faculty, Heinrich-Heine University Düsseldorf, Düsseldorf, Germany

**Keywords:** pediatric oncology, childhood cancer, exercise intervention, immune system, inflammation, natural killer cells, physical performance, physical activity

## Abstract

**Background:**

Pediatric cancer patients are at high risk for life-threatening infections, therapy associated complications and cancer-related side effects. Exercise is a promising tool to support the immune system and reduce inflammation. The primary objective of this systematic review was to evaluate the effects of exercise interventions in pediatric cancer patients and survivors on the immune system.

**Methods:**

For this systematic review (PROSPERO ID: CRD42021194282) we searched four databases (MEDLINE, Cochrane Library, ClinicalTrials.gov, SPORTDiscus) in June 2021. Studies with pediatric patients with oncological disease were included as main criterion. Two authors independently performed data extraction, risk of bias assessment, descriptive analysis and a direction ratio was calculated for all immune cell parameters.

**Findings:**

Of the 1448 detected articles, eight studies with overall n = 400 children and adolescents with cancer and n = 17 healthy children as controls aged 4-19 years met the inclusion criteria. Three randomized, four non-randomized controlled trials and one case series were analyzed descriptively. The exercise interventions had no negative adverse effects on the immune system. Statistically significant results indicated enhanced cytotoxicity through exercise, while changes in immune cell numbers did not differ significantly. Interventions further reduced days of in-hospitalization and reduced the risk of infections. Several beneficial direction ratios in immune parameters were identified favoring the intervention group.

**Interpretation:**

Exercise interventions for pediatric cancer patients and survivors had no negative but promising beneficial effects on the immune system, especially regarding cytotoxicity, but data is very limited. Further research should be conducted on the immunological effects of different training modalities and intensities, during various treatment phases, and for different pediatric cancer types. The direction ratio parameters given here may provide useful guidance for future clinical trials.

**Systemic Review Registration:**

https://www.crd.york.ac.uk/prospero/display_record.php?ID=CRD42021194282, Prospero ID: CRD42021194282.

## Introduction

Children and adolescents with cancer are at high risk for life-threatening infections and therapy associated complications. They further have a risk for disease recurrence or development of secondary malignancies (1, 2). Thus, interventions are needed to decrease the probability of cancer-related side effects and increase the patients’ quality of life. Studies suggest that enhanced immunosurveillance by cytotoxic natural killer (NK) cells and T-cells after moderate exercise might be beneficial for anti-cancer surveillance, as both are highly activated during acute aerobic exercise (3–6). Especially exercise-induced NK cells were recognized to have an anti-tumor effect (7). Furthermore, phagocytosis and oxidative burst in granulocytes are also increased by exercise (8) suggesting that regular exercise strengthens the immune system. Exercise induced effects, like increased NK cell cytotoxicity, lymphocyte proliferation and increased frequencies of granulocytes, have been reported in adult cancer patients (9, 10). Thus, performing moderate exercise interventions regularly might result in fewer infections and better clinical outcome in cancer patients. Growing evidence summarized in systematic reviews suggests positive effects of exercise therapy in pediatric oncology on cardiopulmonary capacity, functional mobility, muscle strength, quality of life and fatigue (11–13). Even though research is still limited, the current studies underline that exercise programs are safe and feasible in children with oncological diseases, while not increasing the risk of mortality, cancer recurrence or side effects (14). Moreover, an in-hospital exercise program seems to reduce days of hospitalization and treatment costs (15). Of note, evidence from experimental murine studies have shown beneficial effects of exercise intervention on graft versus host disease (GvHD) after hematopoietic stem cell transplantation (HSCT) (16, 17).

Studies in adult cancer patients already suggest beneficial effects of exercise on the immune system (9, 10). However, as the immune system undergoes systematic changes from pre- to post-birth (18, 19) and during aging (20, 21), the knowledge taken from the immune response of adult cancer patients after exercise may not be directly transferable to pediatric cancer patients. The type, frequency, duration, and intensity of exercise could also affect the immune system in different ways (22). Therefore, research studies focusing on how exercise affects the pediatric immune system of cancer patients is a relevant research question. The most recent systematic review focusing on general exercise and the immune system including studies with children and adults with cancer has been published in 2013 (10). Thus, this systematic review aims at analyzing the current knowledge on this topic. The primary objective of the review is to determine the effects of exercise interventions in pediatric oncology on the immune system. As secondary objectives the effects on physical and functional performance, body composition, and hematopoietic stem cell transplantation (HSCT) outcome will be discussed.

## Methods

### Search Strategy and Selection Criteria

This systematic review was conducted according to the PRISMA recommendations (23). A comprehensive search by two authors (RB, MG) of four databases (MEDLINE, Cochrane Library, ClinicalTrials.gov, SPORTDiscus) was conducted in June 2021. Search terms were based on PICO (24) for children or adolescents with cancer and/or recipients of stem cell transplantation during and after acute cancer therapy (Patient Population), exercise (Intervention), usual care/healthy control group (Comparator group), and immune function (Outcome). A combination of MeSH terms and search terms in title/abstract and relevant headings, keywords and synonyms for the search focuses was used. There were no restrictions in terms of publication date. The exact search terms are listed in **Supplement 1** and inclusion and exclusion criteria are shown in **Supplement 2**. The protocol of this review was registered in PROSPERO https://www.crd.york.ac.uk/prospero/display_record.php?ID=CRD42021194282.

**Figure 1:**
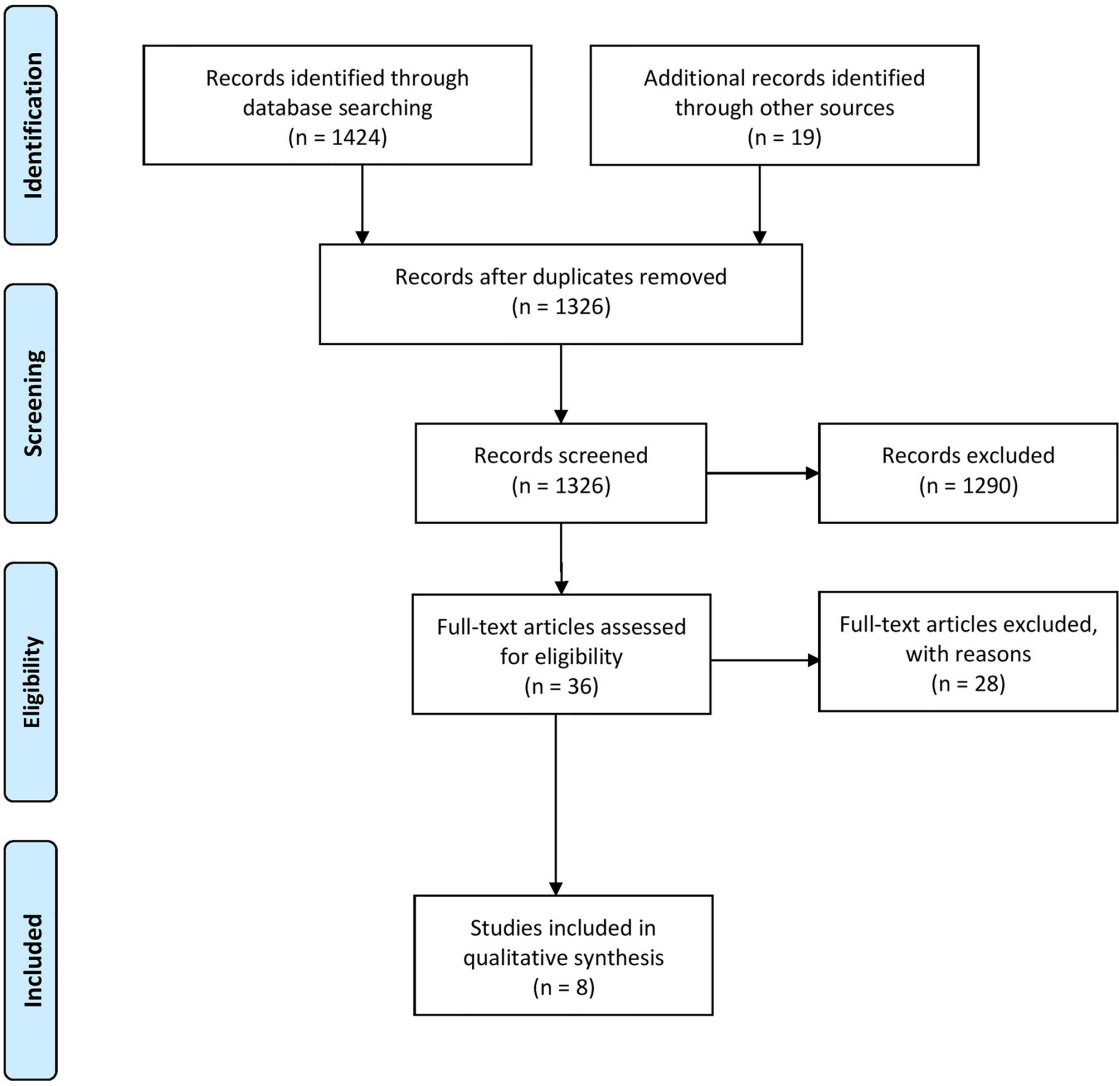
Flow diagram according to Prisma guidelines (25).

After removing duplicates, two authors (RB, SBB) independently reviewed all identified studies by reviewing titles and abstracts. Remaining studies were checked accordingly for eligibility in full text (**Supplement 5**). Disagreements between the reviewers were discussed between the two researchers. If no consensus was reached, a third researcher (MG) was contacted. Eligible studies as well as identified systematic reviews were further screened for potentially missed relevant studies (**Figure 1**).

### Data Analyses

Two authors (RB, SBB) assessed the risk of bias of included studies according to the PEDro scale (26). The PEDro scale is based on the Delphi list and was made for assessment of internal validity and sufficient statistical information (27). This tool is applicable for randomized clinic trials like randomized controlled trials (RCTs) and controlled clinical trial (CCT). The risk of bias was rated on a scale of 0-10 points (**Supplement 3**) and any disagreements were resolved by consensus. Two authors (RB, SBB) independently reviewed all identified studies and extracted relevant information for each study into standardized tables (see **Supplement 4** for details on data extraction and **Table 1** for results). A third researcher (MG) collected this information for each study.

**Table 1:** Characteristics and Results of Included Studies.

Reference number, Registration number and name of register	Study design	Participant characterization	N (IG/CG/HC)	Intervention Description	Frequency (F)/Intensity(I)/Time (T)/Duration (D)/Adherence (A)	Outcome Between-Group Differences (Group)			Outcome Within-Group Differences (Time)			Outcome interaction (group x time)			Direction ratio [Intervention (IG) *vs*. Control (CG) group or healthy control group (HC)]		
**Effects of exercise intervention in context of HSCT on immune parameter**																	
28	Historically controlled study – NCT	Age	20 (7/13/0)	IG:	F: 5x/wk (3 x aerobic, 2 x aerobic & strength)	No significant differences			Not significant after multiple correction: T-lymphocytes (p = .040); CD4^+^ (p = .032); DC`s (p = .001)			Not significant after multiple correction: DC’s (p = .045)			**30d *vs* 15d post-HSCT**:		
		IG: 8 ± 4 yr		Supervised in-hospital strength & aerobic exercises											Leukocytes	IG: 4.1 *vs*. CG: 2.5	↑
		(5-16 yr);													Lymphocytes	IG: 2.5 *vs*. CG: 5.1	↓
		CG: 7 ± 3 yr													Monocytes	IG: 1.4 *vs*. CG: 1.4	→
		(4-13 yr)			I: 50 % - 70 % of age predicted max. HR, hypertrophy training										T-cells	IG: 3.3 *vs*. CG: 5.2	↓
		Cancer entities		CG:											NKs	IG: 1.7 *vs*. CG: 3.5	↓
		Mixed high-risk cancer needing allo-HSCT		Usual care (no training)											NKT	IG: 1.9 *vs*. CG: 4.1	↓
															CD4^+^	IG: 1.9 *vs*. CG: 3.8	↓
															CD8^+^	IG: 3.2 *vs*. CG: 7.4	↓
		Treatment-phase			T: ~50 min										DC`s	IG: 1.3 *vs*. CG: 1.2	→
		On-treatment during allo-HSCT			D: mean ~3 wks												
					(until discharge)												
					A: 92 ± 8% of planned sessions												
29	RCT	Age	6	IG:	F: 3x/wk (1x supervised, 2x home-based)	N/A			N/A			Not significant after multiple correction:			**Pre *vs*. post exercise program**		
		IG: 12.7 ± 4.0 yr (9-17 yr);	(3/3/0)	Supervised in-hospital & home-based strength & aerobic exercises								:		
					I: N/A							Mean ratio			NKs:	IG: 2.2 *vs*. CG: 2.7	→
					T: 60 min							CD56^dim^	(p = .03)				
		CG: 13.3 ± 5.5 yr			D: 10 wks										%		
															NKs	IG: 5.1 *vs*. CG: 2.1	↑
		(8-19 yr)			A: 80.3 ± 1.4%							Ratio:			CD56^dim^	IG: 1.3 *vs*. CG: 1.0	→
				CG:											CD56^bright^	IG: 0.5 *vs*. CG: 1.0	↓
												NKCC	(p <.05)		NKG2D	IG: 1.0 *vs*. CG: 0.9	→
				Usual care (no training)											NK_p_30	IG: 1.2 *vs*. CG: 0.5	↑
		Cancer entities													NK_p_44	IG: 0.4 *vs*. CG: 2.0	↓
		Mixed cancer types after allo-HSCT													pg/ml:		
															IL-2	IG: 2.0 *vs*. CG: 1.0	↑
															IL-4	IG: 2.6 *vs*. CG: 0.8	↑
															IL-6	IG: 3.5 *vs*. CG: 2.3	↑
															IL-8	IG: 4.8 *vs*. CG: 4.1	→
															IL-10	IG: 2.2 *vs*. CG: 1.1	↑
		Treatment-phase													IFNγ	IG: 2.8 *vs*. CG: 0.8	↑
		On-treatment after discharge of allo-HSCT													TNFα	IG: 2.5 *vs*. CG: 0.9	↑
															GM-CSF	IG: 1.8 *vs*. CG: 0.5	↑
30	NCT	Age	118	IG:	F: 5x/wk	Not significant after multiple corrections:			Not significant after multiple corrections:			Not significant after multiple corrections:			**30d *vs* 15d post-HSCT**		
		IG: 11 ± 5 yr (5-18 yr);	(66/54/0)	Supervised in-hospital strength & aerobic exercises	I: 65-80% of age predicted max. HR										allo-HSCT		
															:		
		CG: 10 ± 4 yr (4-18yr)				allo- HSCT			allo- HSCT			allo- HSCT			Leukocytes	IG: 2.8 *vs*. CG: 3.5	→
					T: ~60 min										Neutrophils	IG: 5.5 *vs*. CG: 5.0	→
			allo-HSCT			#:			#:			#:			Lymphocytes	IG: 1.9 *vs*. CG: 2.4	→
					D: ~3 wks	Lymphocytes						EM CD4^+^	(p = .005)		Monocytes	IG: 0.9 *vs*. CG: 1.6	↓
							(p = .045)		Leukocytes	(p <.001)		Naïve CD4^+^	(p = .005)		T-cells	IG: 5.6 *vs*. CG: 1.4	↑
			IG: n = 47	CG:	A: 80.3 ± 1.4%	T-cells	(p = .006)		Neutrophils	(p <.001)		Treg	(p = .003)		actCD4^+^	IG: 1.3 *vs*. CG: 2.8	↓
						actCD4^+^	(p = .004)		Lymphocytes			CD56^bright^	(p = .003)		CM CD4^+^	IG: 1.3 *vs*. CG: 1.7	→
		Cancer entities		Usual care (no training)		CM CD4^+^	(p = .011)			(p = .001)					EM CD4^+^	IG: 1.6 *vs*. CG: 2.0	→
			CG: n= 39			CD56^bright^	(p = .011)		T-cells	(p <.001)					naive CD4^+^	IG: 1.5 *vs*. CG: 2.6	↓
		Mixed high risk cancer types needing allo- or auto-HSCT				CD8^+^	(p = .024)		actCD4^+^	(p <.001)					Treg	IG: 1.0 *vs*. CG: 1.1	→
									CM CD4^+^	(p = .007)		auto-HSCT:			CM CD8^+^	IG: 1.7 *vs*. CG: 1.8	→
						auto-HSCT:			EM CD4^+^	(p <.001)					EM CD8^+^	IG: 11.6 *vs*. CG: 26.3	↓
									Naïve CD4^+^	(p <.001)					Naive CD8^+^	IG: 4.0 *vs*. CG: 1.7	↑
			auto-HSCT												NKs	IG: 3.3 *vs*. CG: 2.4	↑
						:			Treg	(p <.001)		:			CD56^bright^	IG: 4.0 *vs*. CG: 2.0	↑
						Leukocytes	(p = .001)		Naïve CD8^+^	(p <.001)		Naïve CD4^+^		(p = .001)	CD56^dim^	IG: 3.0 *vs*. CG: 3.0	→
						Neutrophils	(p = .048)		NKs	(p = .001)		CD4^+^		(p = .048)	mB-cells	IG: 2.3 *vs*. CG: 0.4	↑
			IG: n = 18			Monocytes	(p = .005)		CD56^bright^	(p <.001)		CD8^+^		(p = .041)	CD4^+^	IG: 1.4 *vs*. CG: 2.0	↓
						T-cells	(p = .037)		CD56^dim^	(p <.001)					CD8^+^	IG: 4.2 *vs*. CG: 3.9	→
		Treatment-phase				Naïve CD4^+^	(p <.001)		mB-cells	(p<.001)					DCs	IG: 0.8 *vs*. CG: 1.2	↓
			CG: n = 14			CD4^+^	(p = .008)		DCs	(p = .034)							
						DCs	(p = .019)										
		On-treatment during allo- or auto-HSCT							Auto-HSCT:						auto-HSCT		
									:						:		
									Lymphocytes								
										(p = .027)					Leukocytes	IG: 1.0 *vs*. CG: 1.5	→
															Neutrophils	IG: 1.2 *vs*. CG: 1.7	
									CM CD4^+^	(p = .040)					Lymphocytes	IG: 0.9 *vs*. CG: 1.5	↓
									EM CD4^+^	(p <.001)					Monocytes	IG: 1.7 *vs*. CG: 1.4	→
									Naïve CD4^+^	(p <.001)					T-cells	IG: 1.3 *vs*. CG: 1.8	→
									Treg	(p = .027)					actCD4^+^	IG: 1.0 *vs*. CG: 1.6	↓
									CD4^+^	(p = .019)					CM CD4^+^	IG: 0.8 *vs*. CG: 1.5	↓
									CD8^+^	(p = .008)					EM CD4^+^	IG: 1.3 *vs*. CG: 0.9	↑
															naive CD4^+^	IG: 1.6 *vs*. CG: 0.9	↑
															Treg	IG: 1.3 *vs*. CG: 2.2	↓
															CM CD8^+^	IG: 1.3 *vs*. CG: 0.4	↑
															EM CD8^+^	IG: 1.3 *vs*. CG: 2.7	↓
															Naive CD8^+^	IG: 2.1 *vs*. CG: 1.5	↑
															NKs	IG: 1.3 *vs*. CG: 1.8	→
															CD56^bright^	IG: 2.6 *vs*. CG: 2.0	→
															CD56^dim^	IG: 0.9 *vs*. CG: 1.6	↓
															mB-cells	IG: 0.8 *vs*. CG: 0.8	→
															CD4^+^	IG: 1.2 *vs*. CG: 1.4	↓
															CD8^+^	IG: 1.5 *vs*. CG: 1.2	→
															DCs	IG: 0.9 *vs*. CG: 0.8	→
31 **;** **ClinicalTrials.gov ID: NCT01575704**	RCT	Age	57 (28/29/0)	IG:	F: 5x/wk	No significant differences			No significant differences			No significant differences			**Post exercise program**		
		IG: 11 yr		Supervised in-hospital strength & aerobic exercises	I: IG: moderate training targeted with RPE (12–14)										N/A ^‡^		
		(5-17 yr)															
		CG: 12 yr	allo-HSCT														
		(6-18 yr)			T: 30-60 min												
			IG: n = 31	CG:	D: ~6 wks (until discharge)												
		Cancer entities		Mental & relaxation training (no training)													
			CG: n = 30		A: IG: 94.4 (63.3-100) %, CG: 68.2 (5.8–100) %												
		Mixed high risk cancer needing allo- or auto-HSCT	auto-HSCT														
			IG: n = 4														
		Treatment-phase On-treatment during allo- or auto-HSCT	CG: n = 5														
**Effects of regular exercise intervention in context of chemotherapy on immune parameter**																	
32 **;**	RCT	Age	20	IG:	F: 3x/wk	Not significant after multiple corrections:			**%:**			Not significant after multiple corrections:			**Pre *vs*. Post exercise program**		
									NKT^*^	(p <.001)				
		IG: 11 ± 4 yr	(9/11/0)	Supervised in-hospital (patient`s room or hospital gym) strength & aerobic exercises	I: 60%–70% of measured max HR, 8-15 rep (hypertrophy training)										#:		
**ClinicalTrials.gov ID: NCT01645436**						%:			Not significant after multiple corrections:			%:			Leukocytes	IG: 0.7 *vs*. CG: 0.7	→
		CG: 12 ± 4 yr				NK 69		(p = .013)				KIR2DS4		(p = .028)	T-cells	IG: 0.6 *vs*. CG: 0.7	→
						NKp44		(p = .021)				NKp46		(p = .037)	B-cells	IG: 0.8 *vs*. CG: 0.3	↑
									%:						NKs	IG: 2.2 *vs*. CG: 1.0	↑
						NKCC (ratio):			CD56^bright^		(p = .017)	pg/ml:			NKT	IG: 1.5 *vs*. CG: 1.3	→
		Cancer entities			T: 60-70 min	Ratio 8:1		(p= .038)	NKp44		(p= .004)	PDGF		(p = .023)	CD56^dim^	IG: 1.8 *vs*. CG: 0.9	↑
															CD56^bright^	IG: 20.0 *vs*. CG: 3.0	↑
					D: 17 wks ± 5										KIR2DL1	IG: 0.5 *vs*. CG: 0.8	↓
		Solid tumors													KIR2DL2/3	IG: 1.0 *vs*. CG: 1.0	→
		(sarcoma, lymphoma, blastoma)			A: 70%										KIR3DL1	IG: 0.5 *vs*. CG: 0.3	↑
															NK 25	IG: 2.0 *vs*. CG: 2.0	→
				CG:											NK 69	IG: 2.0 *vs*. CG: 1.5	↑
															KIR2DS4	IG: 1.7 *vs*. CG: 1.3	↑
															NKG2D	IG: 1.6 *vs*. CG: 1.0	↑
				Usual care (no training)											NKp44	IG: 2.0 *vs*. CG: 1.0	↑
															NKp46	IG: 1.5 *vs*. CG: 1.0	↑
		Treatment-phase													NKp30	IG: 1.7 *vs*. CG: 1.0	↑
		On-treatment during neoadjuvant therapy													DNAM	IG: 2.3 *vs*. CG: 1.3	↑
															NKG2A	IG: 2.0 *vs*. CG: 1.2	↑
															CXCR6	IG: 0.3 *vs*. CG: 1.0	↓
															%:		
															T-cells	IG: 0.8 *vs*. CG: 1.1	→
															B-cells	IG: 0.5 *vs*. CG: 0.3	↑
															NKs	IG: 2.4 *vs*. CG: 1.3	↑
															NKT	IG: 1.0 *vs*. CG: 1.7	↓
															CD56^dim^	IG: 0.9 *vs*. CG: 1.1	→
															CD56^bright^	IG: 2.5 *vs*. CG: 1.8	↑
															KIR2DL1	IG: 0.4 *vs*. CG: 0.6	↓
															KIR2DL2/3	IG: 0.8 *vs*. CG: 1.2	↓
															KIR3DL1	IG: 0.3 *vs*. CG: 0.4	→
															NK 25	IG: 1.1 *vs*. CG: 1.8	↓
															NK 69	IG: 1.3 *vs*. CG: 1.1	→
															KIR2DS4	IG: 1.0 *vs*. CG: 1.8	↓
															NKG2D	IG: 1.0 *vs*. CG: 1.2	→
															NKp44	IG: 1.0 *vs*. CG: 1.7	↓
															NKp46	IG: 1.2 *vs*. CG: 2.9	↓
															NKp30	IG: 1.1 *vs*. CG: 1.3	→
															DNAM	IG: 0.9 *vs*. CG: 1.5	↓
															NKG2A	IG: 1.0 *vs*. CG: 1.3	→
															CXCR6	IG: 1.0 *vs*. CG: 1.8	↓
															NKCC (ratio):		
															8:1	IG: 0.7 *vs*. CG: 0.5	↑
															4:1	IG: 0.8 *vs*. CG: 0.8	→
															2:1	IG: 0.8 *vs*. CG: 0.9	→
															1:1	IG: 0.7 *vs*. CG: 1.0	↓
															pg/ml:		
															CTACK	IG: 1.1 *vs*. CG: 0.9	→
															Eotaxin	IG: 1.0 *vs*. CG: 1.5	↓
															FGF	IG: 1.1 *vs*. CG: 1.3	→
															GSCF	IG: 1.1 *vs*. CG: 0.7	↑
															GRO-α	IG: 1.4 *vs*. CG: 0.7	↑
															HGF	IG: 1.4 *vs*. CG: 1.0	↑
															ICAM	IG: 1.0 *vs*. CG: 0.9	→
															IFNγ	IG: 1.1 *vs*. CG: 1.0	→
															IL-1α	IG: 0.9 *vs*. CG: 0.9	→
															IL-2	IG: 1.1 *vs*. CG: 1.1	→
															IL-3	IG: 0.7 *vs*. CG: 2.0	↓
															IL-4	IG: 0.9 *vs*. CG: 1.0	→
															IL-6	IG: 1.0 *vs*. CG: 0.8	→
															IL-7	IG: 1.3 *vs*. CG: 0.5	↑
															IL-8	IG: 0.5 *vs*. CG: 0.8	↓
															IL-9	IG: 1.0 *vs*. CG: 1.1	→
															IL-10	IG: 0.5 *vs*. CG: 0.6	→
															IL-16	IG: 0.9 *vs*. CG: 0.6	↑
															IL-18	IG: 1.3 *vs*. CG: 0.9	↑
															IP10/CXCL10	IG: 1.9 *vs*. CG: 0.6	↑
															MCP1/CCL2	IG: 1.4 *vs*. CG: 1.0	↑
															MCP3/CCL7	IG: 3.6 *vs*. CG: 0.5	↑
															MIG/CXCL9	IG: 1.6 *vs*. CG: 0.9	↑
															MIF	IG: 1.4 *vs*. CG: 0.5	↑
															MIP-1 α/CCL3	IG: 0.6 *vs*. CG: 0.9	↓
															MSCF	IG: 0.8 *vs*. CG: 1.5	↓
															PDGF	IG: 1.4 *vs*. CG: 0.8	↑
															TNF-β	IG: 0.6 *vs*. CG: 0.8	→
															TRAIL	IG: 1.2 *vs*. CG: 0.8	↑
															VCAM1	IG: 1.1 *vs*. CG: 1.2	→
															VEGF	IG: 0.7 *vs*. CG: 0.8	→
15	NCT	Age	169	IG:	F: 2-3x/wk	No significant differences			#			No significant			**Pre *vs*. Post exercise program**		
		IG: 11 ± 3 yr (4-17 yr);	(68/101/0)	Supervised in-hospital strength & aerobic exercises	I: 65-80% of age predicted max. HR				Leukocytes^*^ (p <.0001)			differences			Leukocytes	IG: 0.6 *vs*. CG: 0.8	↓
		CG: 11 ± 4 yr (4-18yr)			T: ~60-70 min												
		Cancer entities			D: ~22 wks												
		Solid tumor & leukemia		CG:	A: N/A												
				Usual care (no training)													
33	CS	Age	17	IG & HC: Supervised of fitness instructor or parents aerobic exercise	F: 3x/wk	**Initial resting immune function (IG *vs*. HC):**			No significant differences in IG			N/A			**Pre *vs*. Post exercise program**		
		IG: 14 ± 0.6 yr;	(3/3/ 11)		I: 70%–85% of measured max HR,												
		CG: 13.0 ± 3.1 yr;							Significant changes in HC:								
		HC: N/A		CG:	T: 30 min	Leukocytes^*^			Leukocytes^*^						Leukocytes	IG: 0.9 *vs*. HC: 0.8	→
		Cancer entities		Usual care (no training)	D: 12 wks	Lymphocytes^*^			CD3^+*^						Lymphocytes	IG: 0.6 *vs*. HC: 1.3	→
		IG & CG: ALL & neoplasms			A: N/A	CD3^+*^			CD25^+*^						Monocytes	IG: 0.7 *vs*. HC: 1.3	↓
		HC: healthy controls				CD4^+*^									Granulocytes	IG: 0.6 *vs*. HC: 0.8	→
		Treatment-phase				CD8^+*^									T-cells	IG: 0.6 *vs*. HC: 0.8	→
		On Treatment after induction therapy				CD19^+*^									CD4^+^	IG: 0.4 *vs*. HC: 1.1	↓
						CD25^+*^									CD8^+^	IG: 0.7 *vs*. HC: 0.9	→
						PHA-induced lymphocyte proliferation^*^									B-cells	IG: 7.0 *vs*. HC: 1.1	↑
															NKs	IG: 0.8 *vs*. HC: 0.9	→
															CD25^+^	IG: 0.3 *vs*. HC: 0.6	↓
															CD122^+^	IG: 1.3 *vs*. HC: 0.8	↑
															Ratio:		
															CD4^+^/CD8^+^	IG: 0.8 *vs*. HC: 1.1	→
															Cytolytic activity:		
															Spontaneous	IG: 2.3 *vs*. HC: 0.6	↑
															IL-2-induced	IG: 1.3 *vs*. HC: 0.7	↑
															Proliferation:		
															PHA-induced	IG: 0.5 *vs*. HC: 1.1	↓
															PWM-induced	IG: 0.6 *vs*. HC: 1.0	↓
**Effects of acute exercise intervention on immune parameter**																	
34	NCT	Age	10	IG & HC: Supervised in-hospital moderate to vigorous intermittent aerobic exercise	F: N/A	#:			#:			Ratio of active neutrophils compared to unstimulated neutrophils at time 0:			**Pre *vs*. Post exercise intervention**		
			(4/0/6)		I: 70-85% of measured VO2 peak	ALC^*^	(p = .000)		WBC*	(p = .023)					:		
		IG: 11.3 ± 5.3 yr				Eosinophils*			ALC^*^	(p = .008)					WBC	IG: 1.3 *vs*. HC: 1.4	→
		HC: 10.8 ± 4.6 yr					(p = .006)		Monocytes						ALC	IG: 1.5 *vs*. HC: 1.3	→
										(p = .059)					Monocytes	IG: 1.5 *vs*. HC: 1.5	→
					T: 30 min (10 min walk, 10 min run, 10 min walk)							Ratio time15*					
									Eosinophils^*^			(p = .006)			Eosinophils	IG: 1.0 *vs*. HC: 1.1	→
						Ratio of active neutrophils compared to unstimulated neutrophils at time 0:				(p = .042)					Basophils	IG: 1.6 *vs*. HC: 1.9	→
		Cancer entities															
					D: N/A→ acute effects												
		IG: Pre B-ALL													Ratio of active neutrophils compared to unstimulated neutrophils at time 0 (Post *vs*. Pre exercise):		
					A: N/A	Ratio time5^*^			Ratio of active neutrophils:					
		HC: healthy controls					(p = .048)										
									pre-post exercise						Ratio time5	IG: 0.8 *vs*. HC: 0.7	→
						Ratio time10**^◊^**									Ratio time10	IG: 0.6 *vs*. HC: 0.4	→
		Treatment-phase					(p = .074)			(p = .011)					Ratio time15	IG: 0.8 *vs*. HC: 0.6	→
						Ratio time15^*^			post to 1-h post exercise	(p = .045)					**Pre- *vs*. 2-h post exercise intervention**		
							(p = .050)								:		
									1-h to 2-h post exercise						WBC	IG: 1.1 *vs*. HC: 1.2	→
															ALC	IG: 1.1 *vs*. HC: 1.1	→
		On maintenance treatment				Neutrophil oxidative burst:				(p = .052)					Monocytes	IG: 1.3 *vs*. HC: 1.2	→
															Eosinophils	IG: 0.7 *vs*. HC: 0.9	→
						time 0	(p = .029)								Basophils	IG: 1.0 *vs*. HC: 1.6	↓
															Ratio of active neutrophils compared to unstimulated neutrophils at time 0 (2-h post *vs*. Pre exercise):		
															Ratio time 5 min	IG: 0.6 *vs*. HC: 1.2	↓
															Ratio time 10 min	IG: 0.4 *vs*. HC: 1.2	↓
															Ratio time 15 min	IG: 0.5 *vs*. HC: 1.7	↓

Characteristics, exercise descriptions and results from the included studies are summarized. P-values are shown within the columns: Outcome Between-Group Differences (Group), Outcome Within-Group Differences (Time), and Outcome interaction (group x time). A trend ratio was calculated by dividing values 30d post-HSCT by 15d post- HSCT (28, 30), post-HSCT by pre-HSCT (29), posttreatment by baseline (15, 32), final by initial (33), and post- exercise or 2-h post exercise by pre-exercise (34).*, significant (p-values are shown if reported), ◊, significant with p <.1 due to the small sample size of the study; ‡, no calculation of change possible, because only data for one time point is available even after further inquiry; #, cell count, %, percent, ↓↑→, Direction of trend or difference if the ratio of the intervention group differs >30% compared to the ratio of the control/healthy group, ALC, absolute lymphocyte count; ANC, absolute neutrophil count, B, B cells; CD, cluster of differentiation; CG, Control group; CS, case series; DC, dendritic cells; ex., exercise; HC, Healthy controls; HSCT, Hematopoetic stem cell transplantation; IG, Intervention group; IL, Interleukin; KIR, killer cell immunoglobulin-like receptors; min, minute(s); N/A, data not available; NCT, Non-randomized controlled trial; NK, natural killer cells; NK T, natural killer T cells; RCT, Randomized controlled trial; T, T cells; WBC, whole blood cells; wks, weeks; yr, years; h, hour(s); actCD4^+^, activated CD4^+^ T cells; CM CD4^+^, central memory CD4^+^ T cells; EM CD4^+^, effector memory CD4^+^ T cells; Treg, regulatory CD4^+^ T cell; mB-cells, mature B-cells.

For clarity only p-values for significant values are shown in **Table 1**. Due to the limited numbers of studies, different study designs and heterogeneous immunological parameters analyzed, no meta-analysis was conducted. We calculated a direction ratio for each immunological parameter analyzed within the different studies between the start and end point: Direction arrows were given if the ratio of an immunological parameters within the intervention group (IG) differs more than 30% (value IG > 30% control group (CG)/healthy controls) compared to the CG or healthy controls. Calculation and results are shown in **Supplement 4** and **Table 1**.

## Results

In total, 1448 articles were identified in the systematic search, where eight studies were included within this review (**Figure 1**, exclusion criteria listed in **Supplement 2**). Of those, three studies were RCTs (29, 31, 32), four were NCTs (15, 28, 30, 34) and one case series (33). Seven of these studies focused on chronic effects (15, 28–33) of regular exercise and one of them on acute effects after exercise (34). Immune parameters were primary outcomes in five studies (28, 29, 32–34) and secondary outcomes in three studies (15, 30, 31). A total of 400 children within an age range of four to 19 years with different cancer types, such as solid tumor, leukemia, and neoplasms, and 17 children as healthy controls participated in the included studies. 246 boys and 154 girls took part in the included studies, whereas sex is not mentioned in one study (n = 17) (33). Treatment phases and cancer treatment differed within the studies. Detailed study characterizations are available in **Table 1**. Risk of bias assessment could be performed for the three RCTs and studies scored with 4 out of 10 (32), 6 out of 10 (29), and 5 out of 10 (31) possible points (see **Supplement 3**).

Most studies focused on mixed interventions with strength and aerobic exercises. In the study of Chamorro-Vina et al. (28), Morales et al. (30) and Senn-Malashonak et al. (31) participants attended a supervised in-hospital exercise program five times per week for about three weeks until discharge during HSCT. In Chamorro-Vina et al. (29) children performed three times per week a supervised in-hospital & home-based training after discharge of HSCT for ten weeks. In Fiuza-Luces et al. (32) children participated at the supervised in-hospital exercise program three times per week for about 17 weeks on-treatment during neoadjuvant therapy. In Morales et al. (15) participants trained supervised in-hospital two to three times per week for about 22 weeks.

Only in two studies (33, 34) exercise programs consisted of aerobic exercises alone. In Shore and Shepard (33) children participated three times per week for 12 weeks in a supervised aerobic exercise training. In Ladha et al. (34) children attended a supervised in-hospital moderate to vigorous intermittent aerobic exercise test for 30 minutes. Detailed exercise modalities are described in **Table 1**.

### Effects of Regular Exercise Intervention in the Context of HSCT on Immune Parameters

Four studies focused on the immunological changes in the context of HSCT (28–31). Significant effects of the intervention (group x time) were seen for DC counts (p= .045) (28) and following T cell subset counts: effector memory (EM) CD4^+^ (p = .005), regulatory CD4^+^ T cells (Tregs, p = .003), and naïve CD4^+^ T cells (p= .005) during allo-HSCT as well as CD8^+^ T cells (p = .041), naïve CD4^+^ T cells (p= .048), and total CD4^+^ T cell counts (p = .048) during auto-HSCT (30). A significant intervention induced effect (group x time) was noticed for CD56^bright^ NK cell counts in patients receiving allo-HSCT and CD56^dim^ NK cells (mean ratio) showing a significant increase in the IG compared to the CG (p= .003; p = .03) (29, 30). Detected time (within-group) effects within various immune cell subset counts were dominantly seen in patient receiving allo-HSCT, whereas patients receiving auto-HSCT showed more differences in immune cell counts between IG and CG (between- group) (30). All reported significances became non-significant after adjustment for multiple comparison (**Supplement 4**). No further significant intervention induced effect has been noticed for NK cell specific cell surface receptors, such as NKG2D, NKp30, and NKp44.

The NK cells were analyzed for their cytotoxic potential against the target cell line K562 from both groups. A ratio of observed natural killer cytotoxicity (NKCC) pre-training and post-training for groups was calculated. The NKCC was significantly higher in the IG compared to the CG (8 times higher, p <.05) (29). In line with slightly higher cytotoxic NK cell potential, a decreased risk of infections was reported within the IG (30).

Serum cytokines levels were measured for eight different cytokines (**Table 1**). No significant intervention effect was found between the two groups. However, a slight increase of IL-2, IL-4, IFN ***γ***, TNF ***α***, and GM-CSF was observed within the IG, while no change was seen within the CG for the first four cytokines, but a decrease for GM-CSF (29).

### Effects of Regular Exercise Intervention in Context of Chemotherapy on Immune Parameters

Three studies examined changes within the immune system in patients with solid tumors and leukemia receiving chemotherapy (15, 32, 33). No significant interaction (group x time) effect for leukocytes or lymphocyte subset counts was reported. Different dynamics in leukocyte counts were seen for IG and CG during follow-ups unto 5-years after posttreatment (15). From posttreatment and within the 1-year follow-up both groups showed significant decreased leukocyte counts compared to baseline (within-group, all p-values <.001). Within the 2-year follow-up, leukocyte counts in IG differed non-significantly compared to baseline (p = .192), while leukocyte counts remained significantly decreased in CG (p = .011). A slight decrease was also seen within the 3-year follow up in CG (p = .061), but not seen within the 4- and 5-year follow-up (15). The study further reported significantly decreased days of in-hospitalization for the IG compared to CG (p = .031) resulting in reduced treatment costs.

When analyzing various NK cell receptors needed for recognition of HLA-class I molecules and other important NK cell receptors, most receptors showed no intervention effect (group x time), but a trend towards an interaction effect was seen for the KIR2DS4 which remained stable in the IG but increased in the CG (p = .028).

In two studies the cytotoxic potential of the cells were analyzed either by the spontaneous or IL-2-induced cytolytic activity of all mononuclear cells (33) or target-induced NKCC (32), however no significant intervention effect (group x time) was reported. Shore and Shepard (33) showed higher baseline levels of spontaneous as well as IL-2-induced cytolytic activity in the healthy controls compared to the IG (pediatric cancer patients). After exercise, a decrease of spontaneous as well as IL-2-induced cytolytic activity was observed within the healthy controls, while both activities increased in the IG to comparable levels as the healthy controls (33). Fiuza-Luces et al. (2017) reported (32) higher baseline levels of NKCC for all four dilutions in the IG (8:1 – 1:1), which was stable for the next two time points after intervention as well. No significant intervention effect (group x time) was reported for NKCC (32). No significant intervention effect (group x time) was seen for the 31 analyzed inflammatory cytokines after testing for multiple corrections (p = .0016) (32).

### Effects of Acute Exercise Intervention on Immune Parameters

One study focused on the effects on neutrophil count and function after an acute 30-minute exercise intervention (34). IG values were compared to age- and sex-matched healthy controls. Both groups received an acute bout of aerobic exercise and neutrophil counts and function were analyzed pre-exercise, post-exercise, 1h, and 2h post-exercise. Neutrophil oxidative burst was monitored at 0min, 5min, 10min, and 15min (34). The authors observed a significant increase in whole blood cell (WBC, p = .002), absolute neutrophil count (ANC, p = .006), and absolute lymphocyte count (ALC, p = .003) as well as a significant decrease in eosinophils (p = .003) in both groups from pre-exercise to post-exercise. The effects on absolute lymphocyte counts and eosinophils were also significant within the group analyses (ALC, p = .000, eosinophils p = .003). When comparing the oxidative burst capacity of the neutrophils between IG and healthy controls, the study showed a significant higher capacity at 0min within the healthy controls compared to IG (main effect for group, p = .029). After 5min, 10min, and 15min the IG showed a significant higher oxidative burst compared to the healthy controls (p = .048,.078,.050, the study set the significant p-value to p <.1 due to low number of participants).

### Direction Ratios of the immunological Changes

Direction ratios in the context of HSCT with regular exercise intervention showed different immune cell reconstitution dynamics between allo-HSCT and auto-HSCT. In IG compared to CG 30d post-HSCT to 15d post-HSCT either no change or an increase in leukocytes counts were observed. An increase in mature immune cell subsets regarding total NK cell, CD56^bright^ NK cell, total T cell, effector memory (EM) CD8^+^ T cell, and memory B cell counts in patients receiving allo-HSCT was detected, compared to patients with auto-HSCT. Patients with auto-HSCT showed an increase in naïve CD4^+^ and EM CD4^+^ T cells. Both patients’ cohorts showed increased naïve CD8^+^ T cell counts (**Table 1**).

NK cell and NKp30^+^ NK cell frequencies as well as serum cytokine levels of IL-2, IL-4, IL-6, IFN ***γ***, IL-10, TNF ***α***, and GM-CSF were higher in the IG compared to the CG post-exercise intervention compared to pre-exercise intervention after discharge of HSCT.

Total cell counts of NK cells and expression of certain NK cell specific molecules (3DL1, CD69, 2DS4, NKG2A, NKG2D, NKp44, NKp46, NKp30, DNAM) were increased in the IG receiving neoadjuvant therapy compared to the CG after usual care. This was also seen for NK cell frequencies, due to CD56^bright^ NK cells, but not for frequencies of NK-specific molecules which decreased. Furthermore, 13 out of 30 inflammatory serum cytokines levels were increased and five out of 30 decreased (**Table 1**). Two studies showed a direction ratio increase of the IG compared to CG or healthy controls in B cell counts and cytotoxicity (8:1 NKCC or spontaneous cytolytic activity). Besides increase in total lymphocyte counts, monocytes, CD4^+^ T cells, CD25^+^, and CD122^+^ cell counts as well as PHA- and PWM-induced proliferation were decreased between the IG and healthy controls.

For acute exercise intervention effects, a decrease in basophil counts as well as in neutrophil activity ratio time 5, 10, and 15 minutes was detected 2-h post exercise compared to pre-exercise within the IG compared to healthy controls.

### Secondary Outcomes

In children undergoing a HSCT no significant differences in transplantation outcomes such as duration of myelosuppression, neutropenic phase or duration of hospitalization were found between IG and CG (28). In Morales et al., 2020b (30) a trend towards between-group differences in the duration of hospitalization was noted (p = .052) with less days for IG. Same result was reported for children during neoadjuvant or intense chemotherapy treatment period in IG according to Morales et al., 2020a (p = .031) (15). Morales et al., 2020b (30) showed a lower number of infections after allo-HSCT in the exercise group (p = .023 and p = .083 for total and viral infections). Senn-Malashonak et al. (31) and Morales et al., 2020b (30) reported no differences in transplantation outcomes such as complications or GvHD for children undergoing HSCT. The effects on physical and/or functional performance as well as body composition have been reviewed in other studies in depth (13, 14, 35). Hence, this study focused on effects directly related to the immune system.

## Discussion

This systemic review included eight studies evaluating the changes in immune cell parameters after acute and chronic exercise in pediatric cancer patients, survivors, and HSCT recipients. In summary, no negative effects of exercise on the immune system, but positive effects on immune cell functionality, especially cytotoxicity, were noted, whereas immune cell counts, and inflammatory serum cytokine levels showed no significant intervention effect. Notably, some studies reported an exercise induced effect on slightly decreased infection rates and significant decreased days of in-hospitalization resulting in decreased treatment costs.

After acute aerobic exercise, patients with acute lymphoblastic leukemia showed a significant higher oxidative burst in neutrophils compared to healthy controls. This is of particular interest, as neutrophils belong to the innate immune system and are therefore responsible for the first line of defense. Neutrophils are able to migrate into tissues in order to eliminate invading pathogens by reactive oxygen species production, cytokine secretion, and phagocytosis (36). Thus, abnormal low frequencies of neutrophils, called neutropenia, can increase the chance of life-threatening infections and is a common side effect during cancer therapy (37–39). This finding by Ladha et al. (34) is in line with published data from adult cancer patients, where an increase in neutrophil function was observed after acute exercise (40). Studies in adult cancer patients and experimental mouse models further suggest that regular exercise intervention sustains enhanced neutrophil function and migratory capacity (40, 41). Furthermore, exercise can reduce chemotherapy related neutropenia in cancer patients (40) which might decrease the risk of infections in cancer patients. This is in line to a lower number of total and viral infections that could be observed (15).

Regular exercise interventions, comprising of combined aerobic and strength training, in pediatric patients post-HSCT led to significant higher mean ratio of CD56^dim^ NK cells and in patients receiving allo-HSCT also increased T cell, B cell, and NK cell counts. This is in line with current literature, as NK cells are one of the first immune cell subsets to reconstitute after HSCT (42, 43). Besides cytotoxic CD8^+^ T cells, CD56^dim^ NK cells are the only other immune cell subset having cytotoxic potential. Indeed, NKCC was significantly higher (8-fold) within the IG compared to the CG suggesting that exercise increases the functionality of NK cells in pediatric cancer patients, which is comparable with literature in healthy adults (44–46) and adults with cancer (47). This is of major importance post-HSCT, as the reconstituted NK cells have been described to promote a graft-versus-leukemia effect, where the donor derived NK cells prevent a potential relapse (48). High NK cell counts (above a median of 120 per µl blood) at day 32 post-HSCT have been described to predict a higher cumulative event-free survival rate, reduced transplant-related mortality and reduced cumulative relapse incidence (49). Hence, regular exercise in pediatric patients may increase NK cell functionality providing a natural defense against life threatening infections. Of note, within the study by Shore and Shepard (33) which focused on aerobic exercises IG showed a similar cytolytic activity compared to healthy children after exercise, although their initial levels of cytolytic activity were lower. Thus, this exercise induced effect of enhanced cytotoxicity is not only seen in patients receiving HSCT, but also in pediatric cancer patients without HSCT. It has to be mentioned that the type of exercise and exercise intensity also seems to influence NKCC (47). No study conducted in pediatric cancer patients so far focused on NK cell phenotyping and function after acute exercise.

Unfortunately, there is still too little data in pediatric cancer patients to define what exercise type and intensity is most beneficial for this population. Especially as most studies used a combination of aerobic and strength exercise programs. Of note, in healthy adults a recent systematic review highlights that aerobic/endurance training has a greater influence on NK cell cytotoxicity compared to strength training (45). Nevertheless, exercise programs during cancer therapy seem to be a useful and safe tool for children to keep their level of physical or functional performance or to improve muscle strength, physical fitness, body composition and functionality (12, 13, 35, 50). In this regard, guidelines regarding physical activity of pediatric cancer patients have recently been published (51) or are currently under development (52).

In summary, this systematic review suggests that no adverse effects on immunological parameters occurred during or after exercise intervention with pediatric cancer patients. Most studies reported an enhanced functional immune cell capacity after exercise indicating that exercise induces a boost to the immune system within pediatric cancer patients. This is in line with the reported reduced risks of infections and reduced days of in-hospitalization in the IG, which in turn decreases treatment costs. Interestingly, within an up to 5-year follow up, the IG was able to regain a comparable leukocyte count compared to baseline one year earlier compared to the CG, suggesting that exercise induced effects might have long lasting effects. Overall, both acute and chronic exercise interventions seem to enhance immune cell functionality in pediatric cancer patients, however data is limited to one immune cell subset in acute exercise intervention.

Some limitations of this review should be acknowledged. All studies included very limited or very heterogeneous populations. Furthermore, varying exercise interventions, differently composed control groups, and a broad variety of immune parameters made accurate comparison of the studies difficult. Therefore, meta-analyses were not possible and descriptive analyses were used instead. We addressed this issue by including a direction ratio. Finally, assessment of quality was only possible for three RCTs, which have a middle and low-quality rating. This is mainly due to lack of participant blinding, missing intention-to-treat analysis and inadequate follow up. However, most of these difficulties are common in exercise intervention studies. Despite these limitations, the strengths of this review are the systematic and thorough search of several databases and the independent quality assessment by two reviewers. This review provides initial information for the planning and implementation of exercise interventions from an immunological point of view in pediatric oncology, as the included studies indicate that exercise is beneficial for the patients. It further highlights the need of further studies to evaluate and understand the mechanistic effects of different exercise modalities on the immune system in pediatric cancer patients and survivors, especially in the context of allo- and auto-HSCT.

## Data Availability Statement

All data collected for this article, including data extraction tables and the statistical analysis, will be made available. Requests to access these data should be made to the corresponding author.

## Author Contributions

RB: study design, search of four data bases, review of studies, risk of bias assessment, data extraction, data analyses, writing the manuscript. SB: review of studies, risk of bias assessment, data extraction, data analyses, writing the manuscript. MG: study design, search of four data bases, final decision on study inclusion, data collection, writing the manuscript. All authors contributed to the article and approved the submitted version.

## Funding

We acknowledge support by the Open Access Publication Fund of the University of Duisburg-Essen.

## Conflict of Interest

The authors declare that the research was conducted in the absence of any commercial or financial relationships that could be construed as a potential conflict of interest.

## Publisher’s Note

All claims expressed in this article are solely those of the authors and do not necessarily represent those of their affiliated organizations, or those of the publisher, the editors and the reviewers. Any product that may be evaluated in this article, or claim that may be made by its manufacturer, is not guaranteed or endorsed by the publisher.

## Abbreviations

A, Adherence; ALC, Absolute lymphocyte count; ALL, Acute lymphoblastic leukemia; ANC, Absolute neutrophil count; CCT, Controlled clinical trial; CG, Control group; D, Duration; DC, Dendritic cell; EM, Effector memory; F, Frequency; GvHD, Graft-versus-host disease; GvL, Graft-versus-leukemia; HSCT, Hematopoietic stem cell transplantation; I, Intensity; KIR, Killer-cell immunoglobulin-like receptors; IG, Intervention group; MVPA, Moderate-vigorous physical activity; NCT, Non-randomized controlled trials; NK cells, Natural killer cells; NKCC, Natural killer cytotoxicity; NKT, Natural killer T cell; OD, Other designs; RCT, Randomized controlled trials; T, Time; WBC, Whole blood cell.

## References

[1] Berendsen AJ Groot Nibbelink A Blaauwbroek R Berger MY Tissing WJE . Second Cancers After Childhood Cancer–GPs Beware! Scand J Prim Health Care (2013) 31(3):147–52. doi: 10.3109/02813432.2013.824152 PMC3750436 23906108

[2] Schrappe M Bleckmann K Zimmermann M Biondi A Möricke A Locatelli F . Reduced-Intensity Delayed Intensification in Standard-Risk Pediatric Acute Lymphoblastic Leukemia Defined by Undetectable Minimal Residual Disease: Results of an International Randomized Trial (AIEOP-BFM ALL 2000). J Clin Oncol (2018) 36(3):244–53. doi: 10.1200/JCO.2017.74.4946 29148893

[3] Bigley AB Rezvani K Chew C Sekine T Pistillo M Crucian B . Acute Exercise Preferentially Redeploys NK-Cells With a Highly-Differentiated Phenotype and Augments Cytotoxicity Against Lymphoma and Multiple Myeloma Target Cells. Brain Behav Immun (2014) 39:160–71. doi: 10.1016/j.bbi.2013.10.030 24200514

[4] LaVoy EC Bollard CM Hanley PJ Blaney JW O’Connor DP Bosch JA . A Single Bout of Dynamic Exercise Enhances the Expansion of MAGE-A4 and PRAME-Specific Cytotoxic T-Cells From Healthy Adults. Exercise Immunol Rev (2015) 21:144–53. 25826370

[5] Campbell JP Riddell NE Burns VE Turner M van Zanten JJ Drayson MT . Acute Exercise Mobilises CD8+ T Lymphocytes Exhibiting an Effector-Memory Phenotype. Brain Behav Immun (2009) 23(6):767–75. doi: 10.1016/j.bbi.2009.02.011 19254756

[6] Fiuza-Luces C Valenzuela PL Castillo-García A Lucia A . Exercise Benefits Meet Cancer Immunosurveillance: Implications for Immunotherapy. Trends Cancer (2021) 7(2):91–3. doi: 10.1016/j.trecan.2020.12.003 33358110

[7] Idorn M Hojman P . Exercise-Dependent Regulation of NK Cells in Cancer Protection. Trends Mol Med (2016) 22(7):565–77. doi: 10.1016/j.molmed.2016.05.007 27262760

[8] Pedersen BK Hoffman-Goetz L . Exercise and the Immune System: Regulation, Integration, and Adaptation. Physiol Rev (2000) 80(3):1055–81. doi: 10.1152/physrev.2000.80.3.1055 10893431

[9] Bigley AB Simpson RJ . NK Cells and Exercise: Implications for Cancer Immunotherapy and Survivorship. Discovery Med (2015) 19(107):433–45. 26175401

[10] Kruijsen-Jaarsma M Révész D Bierings MB Buffart LM Takken T . Effects of Exercise on Immune Function in Patients With Cancer: A Systematic Review. Exercise Immunol Rev (2013) 19:120–43. 23977724

[11] Stössel S Neu MA Wingerter A Bloch W Zimmer P Paret C . Benefits of Exercise Training for Children and Adolescents Undergoing Cancer Treatment: Results From the Randomized Controlled MUCKI Trial. Front Pediatr (2020) 8:243. doi: 10.3389/fped.2020.00243 32582585 PMC7290004

[12] Braam KI van der Torre P Takken T Veening MA van Dulmen-den Broeder E Kaspers GJ . Physical Exercise Training Interventions for Children and Young Adults During and After Treatment for Childhood Cancer. Cochrane Database Syst Rev (2016) 3(3):Cd008796. doi: 10.1002/14651858.CD008796.pub3 27030386 PMC6464400

[13] Coombs A Schilperoort H Sargent B . The Effect of Exercise and Motor Interventions on Physical Activity and Motor Outcomes During and After Medical Intervention for Children and Adolescents With Acute Lymphoblastic Leukemia: A Systematic Review. Crit Rev Oncol Hematol (2020) 152:103004. doi: 10.1016/j.critrevonc.2020.103004 32580035 PMC8359930

[14] Morales JS Valenzuela PL Rincón-Castanedo C Takken T Fiuza-Luces C Santos-Lozano A . Exercise Training in Childhood Cancer: A Systematic Review and Meta-Analysis of Randomized Controlled Trials. Cancer Treat Rev (2018) 70:154–67. doi: 10.1016/j.ctrv.2018.08.012 30218787

[15] Morales JS Santana-Sosa E Santos-Lozano A Baño-Rodrigo A Valenzuela PL Rincón-Castanedo C . Inhospital Exercise Benefits in Childhood Cancer: A Prospective Cohort Study. Scand J Med Sci Sports (2020) 30(1):126–34. doi: 10.1111/sms.13545 31482597

[16] Fiuza-Luces C González-Murillo A Soares-Miranda L Martínez Palacio J Colmenero I Casco F . Effects of Exercise Interventions in Graft-Versus-Host Disease Models. Cell Transplant (2013) 22(12):2409–20. doi: 10.3727/096368912X658746 23127525

[17] Fiuza-Luces C Soares-Miranda L González-Murillo A Palacio JM Colmenero I Casco F . Exercise Benefits in Chronic Graft Versus Host Disease: A Murine Model Study. Med Sci Sports Exerc (2013) 45(9):1703–11. doi: 10.1249/MSS.0b013e31828fa004 23954992

[18] Olin A Henckel E Chen Y Lakshmikanth T Pou C Mikes J . Stereotypic Immune System Development in Newborn Children. Cell (2018) 174(5):1277–92.e14. doi: 10.1016/j.cell.2018.06.045 30142345 PMC6108833

[19] Bennstein SB Scherenschlich N Weinhold S Manser AR Noll A Raba K . Transcriptional and Functional Characterization of Neonatal Circulating ILCs. Stem Cells Trans Med (2021) N/A:1–16. doi: 10.1002/sctm.20-0300 PMC8133339 33475258

[20] Valiathan R Ashman M Asthana D . Effects of Ageing on the Immune System: Infants to Elderly. Scand J Immunol (2016) 83(4):255–66. doi: 10.1111/sji.12413 26808160

[21] Bennstein SB Weinhold S Manser AR Scherenschlich N Noll A Raba K . Umbilical Cord Blood-Derived ILC1-Like Cells Constitute a Novel Precursor for Mature KIR+NKG2A- NK Cells. eLife (2020) 9:e55232. doi: 10.1101/2020.01.24.918318 32657756 PMC7358013

[22] Schauer T Mazzoni AS Henriksson A Demmelmaier I Berntsen S Raastad T . Exercise Intensity and Markers of Inflammation During and After (Neo-) Adjuvant Cancer Treatment. Endocr Relat Cancer (2021) 28(3):191–201. doi: 10.1530/ERC-20-0507 33608485

[23] Moher D Liberati A Tetzlaff J Altman DG . Preferred Reporting Items for Systematic Reviews and Meta-Analyses: The PRISMA Statement. PloS Med (2009) 6(7):e1000097. doi: 10.1371/journal.pmed.1000097 19621072 PMC2707599

[24] Schardt C Adams MB Owens T Keitz S Fontelo P . Utilization of the PICO Framework to Improve Searching PubMed for Clinical Questions. BMC Med Inf Decision Making (2007) 7(1):16. doi: 10.1186/1472-6947-7-16 PMC1904193 17573961

[25] Page MJ McKenzie JE Bossuyt PM Boutron I Hoffmann TC Mulrow CD . The PRISMA 2020 Statement: An Updated Guideline for Reporting Systematic Reviews. BMJ (2021) 372:n71. doi: 10.1136/bmj.n71 33782057 PMC8005924

[26] Herbert R Moseley A Sherrington C . PEDro: A Database of Randomised Controlled Trials in Physiotherapy. Health Inf Manage (1998) 28(4):186–8. doi: 10.1177/183335839902800410 10387366

[27] Verhagen AP de Vet HC de Bie RA Kessels AG Boers M Bouter LM . The Delphi List: A Criteria List for Quality Assessment of Randomized Clinical Trials for Conducting Systematic Reviews Developed by Delphi Consensus. J Clin Epidemiol (1998) 51(12):1235–41. doi: 10.1016/S0895-4356(98)00131-0 10086815

[28] Chamorro-Viña C Ruiz JR Santana-Sosa E González Vicent M Madero L Pérez M . Exercise During Hematopoietic Stem Cell Transplant Hospitalization in Children. Med Sci Sports Exerc (2010) 42(6):1045–53. doi: 10.1249/MSS.0b013e3181c4dac1 19997035

[29] Chamorro-Viña C Valentín J Fernández L González-Vicent M Pérez-Ruiz M Lucía A . Influence of a Moderate-Intensity Exercise Program on Early NK Cell Immune Recovery in Pediatric Patients After Reduced-Intensity Hematopoietic Stem Cell Transplantation. Integr Cancer Ther (2017) 16(4):464–72. doi: 10.1177/1534735416679515 PMC5739144 27903841

[30] Morales JS González Vicent M Valenzuela PL Castillo-García A Santana-Sosa E Lassaletta A . Tailored Exercise During Hematopoietic Stem Cell Transplantation Hospitalization in Children With Cancer: A Prospective Cohort Study. Cancers (2020) 12(10):3020. doi: 10.3390/cancers12103020 PMC7650695 33080908

[31] Senn-Malashonak A Wallek S Schmidt K Rosenhagen A Vogt L Bader P . Psychophysical Effects of an Exercise Therapy During Pediatric Stem Cell Transplantation: A Randomized Controlled Trial. Bone Marrow Transplant (2019) 54(11):1827–35. doi: 10.1038/s41409-019-0535-z 31089282

[32] Fiuza-Luces C Padilla JR Valentín J Santana-Sosa E Santos-Lozano A Sanchis-Gomar F . Effects of Exercise on the Immune Function of Pediatric Patients With Solid Tumors: Insights From the PAPEC Randomized Trial. Am J Phys Med Rehabil (2017) 96(11):831–7. doi: 10.1097/PHM.0000000000000757 28644246

[33] Shore S Shepard RJ . Immune Responses to Exercise in Children Treated for Cancer. J Sports Med Phys Fitness (1999) 39(3):240–3. 10573667

[34] Ladha A Courneya K Bell G Field C Grundy P . Effects of Acute Exercise on Neutrophils in Pediatric Acute Lymphoblastic Leukemia Survivors: A Pilot Study. J Pediatr Hematol/Oncol (2006) 28:671–7. doi: 10.1097/01.mph.0000243644.20993.54 17023828

[35] Wurz A McLaughlin E Lategan C Ellis K Culos-Reed SN . Synthesizing the Literature on Physical Activity Among Children and Adolescents Affected by Cancer: Evidence for the International Pediatric Oncology Exercise Guidelines (iPOEG). Trans Behav Med (2021) 11(3):699–708. doi: 10.1093/tbm/ibaa136 PMC8033595 33538309

[36] Malech HL Deleo FR Quinn MT . The Role of Neutrophils in the Immune System: An Overview. Methods Mol Biol (Clifton NJ) (2014) 1124:3–10. doi: 10.1007/978-1-62703-845-4_1 PMC6777345 24504942

[37] Celkan T Ozkan A Apak H Diren S Can G Yuksel L . Bacteremia in Childhood Cancer. J Trop Pediatr (2002) 48(6):373–7. doi: 10.1093/tropej/48.6.373 12521283

[38] Goodman M . Managing the Side Effects of Chemotherapy. Semin Oncol Nurs (1989) 5(2 Suppl 1):29–52. doi: 10.1016/0749-2081(89)90080-6 2657931

[39] Orudjev E Lange BJ . Evolving Concepts of Management of Febrile Neutropenia in Children With Cancer. Med Pediatr Oncol (2002) 39(2):77–85. doi: 10.1002/mpo.10073 12116054

[40] Schauer T Hojman P Gehl J Christensen JF . Exercise Training as Prophylactic Strategy in the Management of Neutropenia During Chemotherapy. Br J Pharmacol (2020) 1–13. doi: 10.1111/bph.15141 32449810

[41] Martín-Ruiz A Fiuza-Luces C Rincón-Castanedo C Fernández-Moreno D Gálvez BG Martínez-Martínez E . Benefits of Exercise and Immunotherapy in a Murine Model of Human Non-Small-Cell Lung Carcinoma. Exercise Immunol Rev (2020) 26:100–15. 32139351

[42] Bosch M Khan FM Storek J . Immune Reconstitution After Hematopoietic Cell Transplantation. Curr Opin Hematol (2012) 19(4):324–35. doi: 10.1097/MOH.0b013e328353bc7d 22517587

[43] Dulphy N Haas P Busson M Belhadj S Peffault de Latour R Robin M . An Unusual CD56bright/CD16low NK Cell Subset Dominates the Early Posttransplant Period Following HLA-Matched Hematopoietic Stem Cell Transplantation. J Immunol (2008) 181(3):2227–37. doi: 10.4049/jimmunol.181.3.2227 18641363

[44] Moro-García MA Fernández-García B Echeverría A Rodríguez-Alonso M Suárez-García FM Solano-Jaurrieta JJ . Frequent Participation in High Volume Exercise Throughout Life Is Associated With a More Differentiated Adaptive Immune Response. Brain Behav Immun (2014) 39:61–74. doi: 10.1016/j.bbi.2013.12.014 24384467

[45] Rumpf C Proschinger S Schenk A Bloch W Lampit A Javelle F . The Effect of Acute Physical Exercise on NK-Cell Cytolytic Activity: A Systematic Review and Meta-Analysis. Sports Med (2021) 51(3):519–30. doi: 10.1007/s40279-020-01402-9 PMC7900082 33398798

[46] Llavero F Alejo LB Fiuza-Luces C López Soto A Valenzuela PL Castillo-García A . Exercise Training Effects on Natural Killer Cells: A Preliminary Proteomics and Systems Biology Approach. Exercise Immunol Rev (2021) 27:125–41. 33965896

[47] Zimmer P Schenk A Kieven M Holthaus M Lehmann J Lövenich L . Exercise Induced Alterations in NK-Cell Cytotoxicity - Methodological Issues and Future Perspectives. Exercise Immunol Rev (2017) 23:66–81. 28230531

[48] Arvindam US Aguilar EG Felices M Murphy W Miller J . Chapter 16 - Natural Killer Cells in GvHD and GvL. In: Socié G Zeiser R Blazar BR , editors. Immune Biology of Allogeneic Hematopoietic Stem Cell Transplantation, 2nd ed. Academic Press (2019). p. 275–92.

[49] Huttunen P Taskinen M Siitonen S Saarinen-Pihkala UM . Impact of Very Early CD4+/CD8+ T Cell Counts on the Occurrence of Acute Graft-Versus-Host Disease and NK Cell Counts on Outcome After Pediatric Allogeneic Hematopoietic Stem Cell Transplantation. Pediatr Blood Cancer (2015) 62(3):522–8. doi: 10.1002/pbc.25347 25417898

[50] Santos SDS Moussalle LD Heinzmann-Filho JP . Effects of Physical Exercise During Hospitalization in Children and Adolescents With Cancer: A Systematic Review. Rev Paul Pediatr (2020) 39:e2019313. doi: 10.1590/1984-0462/2021/39/2019313 33027320 PMC7537404

[51] Wurz A McLaughlin E Lategan C Chamorro Viña C Grimshaw SL Hamari L . The International Pediatric Oncology Exercise Guidelines (iPOEG). Trans Behav Med (2021). doi: 10.1093/tbm/ibab028 PMC8604278 34037786

[52] Götte M Gauß G . Bewegungsförderung Und Bewegungstherapie in Der Pädiatrischen Onkologie. Registriernummer 025-036.in preparation (2021).

