# Supplementary material for: Effects of Exercise Interventions on Immune Function in Children and Adolescents With Cancer and HSCT Recipients - A Systematic Review
Source: Front Immunol. 2021 Sep 27;12:746171. doi: 10.3389/fimmu.2021.746171 (PMC8504856; doi:10.3389/fimmu.2021.746171)
Supplement: Supplementary file 1 [file DataSheet_1.docx]

# **Supplement 1. Search terms**

**Pubmed (Title, Abstract)**

(((child[Title/Abstract] OR child*[Title/Abstract] OR adolescen*[Title/Abstract] OR pediatr*[Title/Abstract] OR paediatr*[Title/Abstract] OR juvenil*[Title/Abstract] OR infant[Title/Abstract] OR infant*[Title/Abstract] OR adoles*[Title/Abstract] OR teen[Title/Abstract] OR teen*[Title/Abstract] OR youth[Title/Abstract]) AND (Neoplasm[Title/Abstract] OR neoplasm*[Title/Abstract] OR cancer[Title/Abstract] OR cancer*[Title/Abstract] OR oncolog*[Title/Abstract] OR tumor[Title/Abstract] OR tumor*[Title/Abstract] OR tumour[Title/Abstract] OR tumour*[Title/Abstract] OR leukemia[Title/Abstract] OR leukaemia[Title/Abstract] OR leukaemia*[Title/Abstract] OR leukemia*[Title/Abstract] OR lymphoma[Title/Abstract] OR hodgkin[Title/Abstract] OR non-hodgkin[Title/Abstract] OR sarcoma[Title/Abstract] OR osteosarcoma[Title/Abstract] OR neuroblastoma[Title/Abstract] OR nephroblastoma[Title/Abstract] OR rhabdomyosarcoma[Title/Abstract] OR fibrosarcoma[Title/Abstract] OR hepatom*[Title/Abstract] OR hepatoblastoma[Title/Abstract] OR PNET[Title/Abstract] OR medulloblastoma[Title/Abstract] OR retinoblastoma[Title/Abstract] OR glioma[Title/Abstract] OR teratoma[Title/Abstract] OR myeloproliferative disease[Title/Abstract] OR myelodysplastic syndrome[Title/Abstract] OR ependymoma[Title/Abstract] OR astrocytoma[Title/Abstract] OR carcinoma[Title/Abstract] OR germinoma[Title/Abstract] OR dysgerminoma[Title/Abstract] OR bone marrow transplant*[Title/Abstract] OR stem cell transplant*[Title/Abstract] OR AML[Title/Abstract] OR hematoonco*[Title/Abstract] OR haematoonco*[Title/Abstract] OR haemato oncological[Title/Abstract] OR haemato-oncological[Title/Abstract] OR hemato oncological[Title/Abstract] OR hemato-oncological[Title/Abstract] OR childhood ALL[Title/Abstract] OR T-cell[Title/Abstract] OR B-cell[Title/Abstract] OR ewing[Title/Abstract] OR blastoma[Title/Abstract])) AND (sport*[Title/Abstract] OR sport[Title/Abstract] OR exercise[Title/Abstract] OR exercis*[Title/Abstract] OR physical activity[Title/Abstract] OR active video gaming[Title/Abstract] OR exergaming[Title/Abstract] OR yoga[Title/Abstract] OR pilates[Title/Abstract] OR move*[Title/Abstract] OR movement therapy[Title/Abstract] OR training[Title/Abstract] OR work out[Title/Abstract] OR aerobic*[Title/Abstract] OR running[Title/Abstract] OR walk*[Title/Abstract] OR physical fitness[Title/Abstract] OR sport therapy[Title/Abstract] OR endurance[Title/Abstract] OR strength[Title/Abstract] OR physical therapy[Title/Abstract] OR physical therapies[Title/Abstract] OR physiotherapy[Title/Abstract] OR physiotherapies[Title/Abstract] OR gymnastic*[Title/Abstract] OR cycling[Title/Abstract] OR fitness[Title/Abstract] OR motor activity[Title/Abstract])) AND (immun*[Title/Abstract] OR NK cells[Title/Abstract] OR lymphocyte*[Title/Abstract] OR killer cell*[Title/Abstract] OR NK cells[Title/Abstract] OR natural killer cells[Title/Abstract] OR NK-cells[Title/Abstract] OR innate lymphoid cell[Title/Abstract] OR lymphocyte[Title/Abstract] OR cytokine[Title/Abstract] OR chemokines[Title/Abstract] OR interferon[Title/Abstract] OR interferons[Title/Abstract] OR IFN[Title/Abstract] OR interleukin[Title/Abstract] OR tumor necrosis factor[Title/Abstract] OR tumour necrosis factor[Title/Abstract] OR TNF[Title/Abstract] OR macrophage[Title/Abstract] OR neutrophil[Title/Abstract] OR mast cells[Title/Abstract] OR eosinophil[Title/Abstract] OR white blood cells[Title/Abstract] OR WBCs[Title/Abstract] OR leukocytes[Title/Abstract] OR leucocytes[Title/Abstract] OR granulocytes[Title/Abstract] OR neutrophils[Title/Abstract] OR eosinophils[Title/Abstract] OR basophil[Title/Abstract] OR monocytes[Title/Abstract] OR B-cells[Title/Abstract] OR T-cells[Title/Abstract] OR toll-like receptors[Title/Abstract] OR cytosolic receptors[Title/Abstract] OR inflamma*[Title/Abstract] OR phagocytes[Title/Abstract] OR dentritic cells[Title/Abstract] OR Killer T cells[Title/Abstract] OR helper T cells[Title/Abstract] OR gamma delta T cells[Title/Abstract] OR B cells[Title/Abstract] OR T cells[Title/Abstract] OR Tregs[Title/Abstract] OR mastocyte[Title/Abstract] OR labrocyte[Title/Abstract] OR NKT[Title/Abstract])

**SportDiscus (Title, Abstract)**

TI ( (child OR child* OR adolescen* OR pediatr* OR paediatr* OR juvenil* OR infant OR infant* OR adoles* OR teen OR teen* OR youth ) AND TI ( Neoplasm OR neoplasm* OR cancer OR cancer* OR oncolog* OR tumor OR tumor* OR tumour OR tumour* OR leukemia OR leukaemia OR leukaemia* OR leukemia* OR lymphoma OR hodgkin OR non-hodgkin OR sarcoma OR osteosarcoma OR neuroblastoma OR nephroblastoma OR rhabdomyosarcoma OR fibrosarcoma OR hepatom* OR hepatoblastoma OR PNET OR medulloblastoma OR retinoblastoma OR glioma OR teratoma OR myeloproliferative disease OR myelodysplastic syndrome OR ependymoma OR astrocytoma OR carcinoma OR germinoma OR dysgerminoma OR bone marrow transplant* OR stem cell transplant* OR AML OR hematoonco* OR haematoonco* OR haemato oncological OR haemato-oncological OR hemato oncological OR hemato-oncological OR childhood ALL OR T-cell OR B-cell OR ewing OR blastoma ) AND TI ( sport* OR sport OR exercise OR exercis* OR physical activity OR active video gaming OR exergaming OR yoga OR pilates OR move* OR movement therapy OR training OR work out OR aerobic* OR running OR walk* OR physical fitness OR sport therapy OR endurance OR strength OR physical therapy OR physical therapies OR physiotherapy OR physiotherapies OR gymnastic* OR cycling OR fitness OR motor activity ) AND TI ( immun* OR NK cells OR lymphocyte* OR killer cell* OR NK cells OR natural killer cells OR NK-cells OR innate lymphoid cell OR lymphocyte OR cytokine OR chemokines OR interferon OR interferons OR IFN OR interleukin OR tumor necrosis factor OR tumour necrosis factor OR TNF OR macrophage OR neutrophil OR mast cells OR eosinophil OR white blood cells OR WBCs OR leukocytes OR leucocytes OR granulocytes OR neutrophils OR eosinophils OR basophil OR monocytes OR B-cells OR T-cells OR toll-like receptors OR cytosolic receptors OR inflamma* OR phagocytes OR dentritic cells OR Killer T cells OR helper T cells OR gamma delta T cells OR B cells OR T cells OR Tregs OR mastocyte OR labrocyte OR NKT) ) OR AB ( (child OR child* OR adolescen* OR pediatr* OR paediatr* OR juvenil* OR infant OR infant* OR adoles* OR teen OR teen* OR youth ) AND AB ( Neoplasm OR neoplasm* OR cancer OR cancer* OR oncolog* OR tumor OR tumor* OR tumour OR tumour* OR leukemia OR leukaemia OR leukaemia* OR leukemia* OR lymphoma OR hodgkin OR non-hodgkin OR sarcoma OR osteosarcoma OR neuroblastoma OR nephroblastoma OR rhabdomyosarcoma OR fibrosarcoma OR hepatom* OR hepatoblastoma OR PNET OR medulloblastoma OR retinoblastoma OR glioma OR teratoma OR myeloproliferative disease OR myelodysplastic syndrome OR ependymoma OR astrocytoma OR carcinoma OR germinoma OR dysgerminoma OR bone marrow transplant* OR stem cell transplant* OR AML OR hematoonco* OR haematoonco* OR haemato oncological OR haemato-oncological OR hemato oncological OR hemato-oncological OR childhood ALL OR T-cell OR B-cell OR ewing OR blastoma ) AND AB ( sport* OR sport OR exercise OR exercis* OR physical activity OR active video gaming OR exergaming OR yoga OR pilates OR move* OR movement therapy OR training OR work out OR aerobic* OR running OR walk* OR physical fitness OR sport therapy OR endurance OR strength OR physical therapy OR physical therapies OR physiotherapy OR physiotherapies OR gymnastic* OR cycling OR fitness OR motor activity ) AND AB ( immun* OR NK cells OR lymphocyte* OR killer cell* OR NK cells OR natural killer cells OR NK-cells OR innate lymphoid cell OR lymphocyte OR cytokine OR chemokines OR interferon OR interferons OR IFN OR interleukin OR tumor necrosis factor OR tumour necrosis factor OR TNF OR macrophage OR neutrophil OR mast cells OR eosinophil OR white blood cells OR WBCs OR leukocytes OR leucocytes OR granulocytes OR neutrophils OR eosinophils OR basophil OR monocytes OR B-cells OR T-cells OR toll-like receptors OR cytosolic receptors OR inflamma* OR phagocytes OR dentritic cells OR Killer T cells OR helper T cells OR gamma delta T cells OR B cells OR T cells OR Tregs OR mastocyte OR labrocyte OR NKT) )

**Cochrane (Title, Abstract, Keyword)**

(child OR child* OR adolescen* OR pediatr* OR paediatr* OR juvenil* OR infant OR infant* OR adoles* OR teen OR teen* OR youth) in Title Abstract Keyword AND (Neoplasm OR neoplasm* OR cancer OR cancer* OR oncolog* OR tumor OR tumor* OR tumour OR tumour* OR leukemia OR leukaemia OR leukaemia* OR leukemia* OR lymphoma OR hodgkin OR non-hodgkin OR sarcoma OR osteosarcoma OR neuroblastoma OR nephroblastoma OR rhabdomyosarcoma OR fibrosarcoma OR hepatom* OR hepatoblastoma OR PNET OR medulloblastoma OR retinoblastoma OR glioma OR teratoma OR “myeloproliferative disease” OR “myelodysplastic syndrome” OR ependymoma OR astrocytoma OR carcinoma OR germinoma OR dysgerminoma OR “bone marrow transplant*” OR “stem cell transplant*” OR AML OR hematoonco* OR haematoonco* OR “haemato oncological” OR haemato-oncological OR “hemato oncological” OR hemato-oncological OR “childhood ALL” OR T-cell OR B-cell OR ewing OR blastoma) in Title Abstract Keyword AND (sport* OR sport OR exercise OR exercis* OR “physical activity” OR “active video gaming” OR exergaming OR yoga OR pilates OR move* OR “movement therapy” OR training OR “work out” OR aerobic* OR running OR walk* OR “physical fitness” OR “sport therapy” OR endurance OR strength OR “physical therapy” OR “physical therapies” OR physiotherapy OR physiotherapies OR gymnastic* OR cycling OR fitness OR “motor activity”) in Title Abstract Keyword AND (immun* OR “NK cells” OR lymphocyte* OR “killer cell*” OR “NK cells” OR “natural killer cells” OR NK-cells OR “innate lymphoid cell” OR lymphocyte OR cytokine OR chemokines OR interferon OR interferons OR IFN OR interleukin OR “tumor necrosis factor” OR “tumour necrosis factor” OR TNF OR macrophage OR neutrophil OR “mast cells” OR eosinophil OR “white blood cells” OR WBCs OR leukocytes OR leucocytes OR granulocytes OR neutrophils OR eosinophils OR basophil OR monocytes OR B-cells OR T-cells OR “toll-like receptors” OR “cytosolic receptors” OR inflamma* OR phagocytes OR “dentritic cells” OR “Killer T cells” OR “helper T cells” OR “gamma delta T cells” OR “B cells” OR “T cells” OR Tregs OR mastocyte OR labrocyte OR NKT) in Title Abstract Keyword

# **Supplement 2. Criteria for inclusion and exclusion of the studies**

| **Inclusion** | **Exclusion** | **Reasons for exclusion** |
| --- | --- | --- |
| (1) Participants were patients and survivors of cancer and / or recipients of hematopoietic stem cell transplantation (HSCT) aged ≤ 18 years at diagnosis and < 21 years at study participation.  (2) Outcome was a parameter of immune cells and / or their function.  (3) Participants of intervention group were included in any exercise intervention.  (4) Peer reviewed randomized and non-randomized controlled trials.  (5) Studies with more than five patients in total.  (6) Published in German or English. | (1) Not childhood cancer patients or survivor or recipient of HSCT (population should be diagnosed <18 years, age at study <21 years)  (2) Endpoint is not parameter of immune function  (3) No physical activity intervention  (4) No original article (abstracts, reviews, posters, case reports, commentaries or letters without own results are excluded)  (5) Not peer-reviewed article  (6) No information on the content we need (old articles)  (7) Duplicate study not identified before  (8) Less than 5 participants | (1) Focus on children and adolescent cancer patients  (2 + 3) Focus is on how exercise intervention effects the immune system  (4 + 5) For data extraction the original data taken directly from the articles was needed  (6) Without the data, no data extraction is possible  (7) already included  (8) Fewer than 5 participants will lead to no possible statistical outcomes or trends |

# **Supplement 3. Quality assessment using PEDro scale.**

| **Study** | **Eligibility criteria*** | **Random allocation** | **Concealed allocation** | **Baseline comparability** | **Blind subjects** | **Blind therapists** | **Blind assesors** | **Adequate follow-up** | **Intention-to-treat analysis** | **Between-group comparisons** | **Point estimates and variability** | **Total score** |
| --- | --- | --- | --- | --- | --- | --- | --- | --- | --- | --- | --- | --- |
| **Fiuza-Luces et al. 2017** | No | Yes | No | No | No | No | Yes | No | No | Yes | Yes | 4/10 |
| **Chamorro-Vina et al. 2017** | Yes | Yes | Yes | Yes | No | No | Yes | No | No | Yes | Yes | 6/10 |
| **Senn-Malashonak et al. 2019** | Yes | Yes | No | Yes | No | No | No | No | Yes | Yes | Yes | 5/10 |

** Eligibility criteria item does not contribute to total score*

# **Supplement 4. Defined variables for which data were extracted and any summary measures used**

| General information | Intervention | Primary outcome of immunological parameter |
| --- | --- | --- |
| (1) Study details: authors, year of publication, study design  (2) Patient characteristics: age at study, cancer type and stage, treatment phase  (3) Sample size  (4) Intervention description of intervention group (IG) and Control group (CG) or healthy control group | (5) Intervention details: frequency, intensity, time, duration, adherence. | (6) Outcome between-group differences (group)  (7) Outcome within-group differences (time)  (8) Outcome interaction (group x time)  (9) Direction of the calculated ratio focusing on leukocyte/ lymphocyte counts, lymphocyte subset counts  Calculation of change for studies in context of HSCT ^27, 29^: 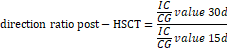 and for studies in context of chemotherapy ^24, 25, 28, 30, 31^: 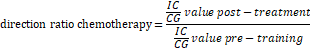 Due to the lack of pre-training values, this could not be done for one study ^26^. A trend arrow was set if the direction ratio was < or > than 30%. |

# **Supplement 5. List of studies excluded at full-text screening stage, with brief reasons**

| **Author, Year** | **Reason for exclusion** |
| --- | --- |
| Bee et al., 2013 | not original article (abstracts, reviews, posters, commentaries, case reports or letters without own results are excluded) |
| Kast et al., 2018 | not childhood cancer patient or survivor or recipient of HSCT (population should be diagnosed <18 years, age at study <21 years); not original article (abstracts, reviews, posters, commentaries, case reports or letters without own results are excluded) |
| Blöte et al., 2019 | no physical activity intervention |
| Chamorro-Viña et al., 2012 | not original article (abstracts, reviews, posters, commentaries, case reports or letters without own results are excluded) |
| Chang et al., 2015 | no physical activity intervention |
| Chinapaw et al., 2012 | endpoint is not parameter of immune function; not original article (abstracts, reviews, posters, commentaries, case reports or letters without own results are excluded) |
| Duncan et al., 2019 | not childhood cancer patient or survivor or recipient of HSCT (population should be diagnosed <18 years, age at study <21 years); |
| Heuvel et al., 2017 | not childhood cancer patient or survivor or recipient of HSCT (population should be diagnosed <18 years, age at study <21 years) |
| Hossain et al., 2020 | no physical activity intervention; not childhood cancer patient or survivor or recipient of HSCT (population should be diagnosed <18 years, age at study <21 years) |
| Soares-Miranda et al., 2013 | not original article (abstracts, reviews, posters, commentaries, case reports or letters without own results are excluded) |
| Kunin-Batson et al., n.a. | not original article (abstracts, reviews, posters, commentaries, case reports or letters without own results are excluded) |
| Ness et al., 2015 | not original article (abstracts, reviews, posters, commentaries, case reports or letters without own results are excluded) |
| Senn-Malashonak et al., 2014 | not original article (abstracts, reviews, posters, commentaries, case reports or letters without own results are excluded) |
| Soares-Miranda et al., 2013 | duplicate study not identified before; not original article (abstracts, reviews, posters, commentaries, case reports or letters without own results are excluded) |
| West et al., 2014 | not original article (abstracts, reviews, posters, commentaries, case reports or letters without own results are excluded) |
| Kabak et al., 2016 | endpoint is not parameter of immune function |
| Ruiz et al., 2010 | endpoint is not parameter of immune function |
| San Juan et al., 2007b | endpoint is not parameter of immune function |
| San Juan et al., 2007a | endpoint is not parameter of immune function |
| Ladha et al., 2006 | duplicate study not identified before |
| Warner et al., 1998 | endpoint is not parameter of immune function |
| Reilly et al., 1998 | endpoint is not parameter of immune function |
| Aznar et al., 2006 | endpoint is not parameter of immune function |
| Robertson et al., 2009 | endpoint is not parameter of immune function;  not original article (abstracts, reviews, posters, commentaries, case reports or letters without own results are excluded) |
| San Juan et al., 2007c | endpoint is not parameter of immune function;  duplicate study not identified before |
| Rosenhagen et al., 2011 | endpoint is not parameter of immune function |
| Radom-Aizik et al., 2013 | not childhood cancer patient or survivor or recipient of HSCT (population should be diagnosed <18 years, age at study <21 years) |
| Kurpiers et al., 2019 | not original article (abstracts, reviews, posters, commentaries, case reports or letters without own results are excluded) |
